# Evaluation of Cardiovascular Risk Factors Among Adults With Perinatally Acquired HIV

**DOI:** 10.1093/ofid/ofaf629

**Published:** 2025-11-06

**Authors:** Merle Henderson, Vibeke Klastrup, Salwa Ahmad, Jessica Glenn, Sara Ayres, Hana Jadayel, Paula Seery, Caroline Foster, Sarah Fidler

**Affiliations:** Department of Infectious Diseases, Imperial College London, London, UK; 900 Clinic, Imperial College Healthcare Trust, London, UK; Department of Surgery and Cancer, Imperial College NIHR BRC, London, UK; Department of Clinical Medicine, Aarhus University, Aarhus, Denmark; Department of Infectious Diseases, Aarhus University Hospital, Aarhus, Denmark; Department of Infectious Diseases, Imperial College London, London, UK; Department of Infectious Diseases, Imperial College London, London, UK; 900 Clinic, Imperial College Healthcare Trust, London, UK; 900 Clinic, Imperial College Healthcare Trust, London, UK; 900 Clinic, Imperial College Healthcare Trust, London, UK; Department of Infectious Diseases, Imperial College London, London, UK; 900 Clinic, Imperial College Healthcare Trust, London, UK; Department of Infectious Diseases, Imperial College London, London, UK; 900 Clinic, Imperial College Healthcare Trust, London, UK; Department of Surgery and Cancer, Imperial College NIHR BRC, London, UK

**Keywords:** cardiovascular risks, hypertension, metabolic syndrome, PDAY score, perinatally acquired HIV

## Abstract

**Background:**

Despite successful ART, people with HIV are at increased risk of non–AIDS-related comorbidities, including cardiovascular and metabolic disease. Adults with perinatally acquired HIV (PaHIV) may face additional risks due to lifelong HIV-related inflammation and ART exposure. We explored cardiovascular and metabolic risk factors in a cohort of adults with PaHIV.

**Methods:**

Case-note review of adults with PaHIV ≥18 years attending a UK specialist service. Hypertension was defined by World Health Organisation (WHO; ≥ 140/90 mmHg) and American Heart Association (AHA; ≥ 130/80 mmHg) guidelines. Standard lipid and blood pressure thresholds defined metabolic syndrome [triglycerides ≥1.7 mmol/L, high-density lipoprotein <1.04 mmol/L (men) and <1.29 mmol/L (women), BP ≥130/85 mmHg]. CVD risk was assessed using modifiable factors and Pathobiological Determinants of Atherosclerosis in Youth (PDAY) scores for coronary arteries (CAs) and abdominal aorta (AA).

**Results:**

The cohort included 225 adults with PaHIV; median age 27 (IQR 23, 30) years, 55% female, and 86% Black ethnicity. Median CD4 count 634 (IQR 438, 815) cells/μL and ART duration 19 (IQR 13, 22) years. About 83% had HIV-1 RNA <50 copies/mL. Hypertension was identified in 9% and 21% of participants by WHO and AHA criteria, respectively. Metabolic syndrome was present in 3%. Elevated PDAY scores ≥1 were observed in 57% for CA and 51% for AA.

**Conclusions:**

Despite viral suppression, over half the cohort had elevated PDAY scores, predictive of increased cardiovascular risk. WHO-defined hypertension rates were similar to an age-matched UK population; however, 1 in 5 were hypertensive by AHA criteria. Statin initiation guidelines may need adaptation for this population.

Antiretroviral therapy (ART) has transformed survival for people with HIV-1, preventing onward transmission and leading to a substantial reduction in HIV-related morbidity and mortality, normalizing life expectancy for most [1, 2]. Highlighting the success of ART, many children born with HIV are now transitioning into adulthood, with the oldest already in their fifth decade [3]. However, despite effective ART, non–AIDS-related conditions remain a leading cause of death among people with HIV, with cardiovascular diseases (CVDs) being the most prevalent [4].

CVDs, including stroke and heart attacks, are the leading causes of death worldwide, making preventative strategies for these conditions essential [5]. Risk factors for CVDs include hypertension and hypercholesterolemia, both of which are modifiable conditions [6]. Among age-matched individuals in the general population in the United Kingdom (UK) aged 16–44 years, the rates of hypertension and hypercholesterolemia are approximately 10% and 50%, respectively [7]. In the United States (US), where more stringent criteria for hypertension are applied [blood pressure (BP) ≥130/80 mmHg], the rate of hypertension in the same age group is around 22% [8].

People with HIV have an increased risk of CVDs, when compared to populations without HIV [9, 10]. The mechanisms underlying this increased risk are likely multifactorial, related to both traditional (eg, male sex, physical inactivity, poor diet, and smoking) and HIV-specific risk factors for CVD [eg, viral transcription despite suppressed plasma viraemia, chronic immune activation and inflammation, and ART-related toxicities, with previous exposure to boosted protease inhibitors (PIs) and use of the nucleoside reverse transcriptase inhibitors (NRTIs) stavudine, didanosine, and abacavir, associated with a higher risk of CVD] [11–13]. It can be hypothesized that these risk factors may be more pronounced in adults with perinatally-acquired HIV (PaHIV), who have experienced lifelong exposure to HIV and increasingly to ART, including during immune maturation [14]. Additionally, lifestyle and social factors that disproportionately affect the cohort of adults with PaHIV may exacerbate these biological risks, including migration, poverty, smoking, substance use, and mental health issues [14–16].

Traditional CVD risk tools emphasize chronological age, potentially underestimating CVD risk in people with HIV, especially young adults with PaHIV. The Pathobiological Determinants of Atherosclerosis in Youth (PDAY) score is a validated risk assessment tool used to estimate the risk of atherosclerotic lesions in young adults in the coronary artery (CA) and abdominal aorta (AA). The PDAY scoring system includes traditional risk factors for CVD and was developed based on autopsy data from young adults aged 15–34 years without HIV. In the original PDAY study, CA and AA scores ≥1 were linked to an 18% (95% CI: 14, 22) and 29% (95% CI: 23, 35) higher odds of advanced atherosclerotic lesions, respectively [17]. A study on cardiovascular risk in US adolescents (median age, 16.7 years) with PaHIV revealed that a substantial proportion had a PDAY score ≥1 (48% CA and 24% AA scores ≥1), suggestive of an increased CVD risk [11]. Key predictors of CA scores ≥1 included male sex, a history of an AIDS-defining condition, prolonged use of ritonavir-boosted PI, and no previous tenofovir use. Key predictors of AA scores ≥1 included suppressed viral load and a longer duration of boosted PI use. The association with viral suppression may reflect toxicity related to ART exposure. These findings were reflected in a recent U.S. cohort study, which demonstrated high rates of non–AIDS-related comorbidities in adults with PaHIV aged 18–30 years, including type 2 diabetes mellitus, hyperlipidemia, hypertension, and chronic kidney disease [18].

Building on these recent data, we aimed to assess the prevalence of hypertension and metabolic syndrome in a unique U.K. cohort of adults with PaHIV and estimate cardiovascular risk using PDAY scores for the CA and the AA.

## METHODS

### Study Design

This study was a retrospective electronic case-note review using data from a UK adult PaHIV service, approved as a clinical audit. The project was registered as a service evaluation with the Imperial College Healthcare NHS Trust (ICHT) NHS audit team. In line with UK Health Research Authority guidance, formal ethical approval was not required, as all data were routinely collected during clinical care and recorded in an anonymized, password-protected database [19]. Participants were adults with PaHIV aged 18–40 years. Viral suppression was defined as plasma HIV RNA <50 copies/mL, and HIV viremia as ≤50 copies/mL at the time of data collection. Sociodemographic variables were taken from clinic registration data, ART history from cumulative treatment records, and comorbidities/clinical biomarkers from the most recent clinic visit documented at the time of data collection. ART duration was calculated from first initiation in childhood through to the date of data collection, including pediatric history where available. Regimen switches were frequent, but only cumulative duration and class exposure were recorded. The primary outcomes were prevalence of hypertension, metabolic syndrome, and elevated PDAY scores.

Hypertension was defined according to both the World Health Organisation (WHO) and the American Heart Association (AHA) guidelines. WHO defines hypertension as an in-clinic BP ≥140/90 mmHg across the last 3 attendances, while AHA defines it as ≥130/80 mmHg across the last 3 attendances. Metabolic syndrome was defined as triglycerides ≥1.7 mmol/L, high-density lipoprotein (HDL) < 1.04 mmol/L for men and <1.29 mmol/L for women, and BP ≥130/85 mmHg [20]. Fasting glucose and waist circumference data were unavailable.

The PDAY risk scores for CA and AA were calculated using established algorithms that estimate the probability of advanced atherosclerotic lesions based on modifiable risk factors [17]. These include non–high-density lipoprotein (HDL) cholesterol, HDL cholesterol, smoking, hypertension, obesity defined by body mass index (BMI) > 30 kg/m^2^, and glycemic control. Individuals were considered smokers if they reported active tobacco use. Hypertension was defined as an in-clinic BP ≥140/90 mmHg across the last 3 attendances. Hyperglycemia was defined as glycated hemoglobin (HbA1c) > 8%.

### Data Extraction

The collected variables included age, gender, ethnicity, height, weight, smoking status, last absolute CD4+ T cell count, nadir CD4+ T cell count, where available, HIV viral load, current and previous ART, years on ART, Center for Disease Control and Prevention (CDC) clinical classification for HIV infection [A, not/mildly symptomatic; B, moderately symptomatic; C, severely symptomatic (AIDS definition)], past AIDS-defining illnesses, total cholesterol, triglycerides, HDL-C, LDL-C, non-HDL, HbA1c, in-clinic BP from the last 3 visits, BP investigations, and antihypertensive medications.

### Statistical Analysis

Data are presented as medians [interquartile ranges (IQR)] or counts (percentages). The Wilcoxon rank-sum test was used to calculate *P* values for continuous data, while Fisher's exact test and Pearson's Chi-square test were used for categorical variables. Statistical analyses for CA and AA risk scores were performed separately. High risk was defined as a PDAY score ≥1. A multivariable logistic regression model included variables associated with high-risk scores in univariable analysis (*P* ≤ .10). Among highly correlated univariable predictors, the variable with the higher univariable c-statistic was retained in the multivariable model to reduce collinearity and improve model stability. Analyses were conducted on available data using a complete case approach; no imputation of missing values was performed. *P* values ≤.05 were considered statistically significant. We used Rversion 4.4.0 (2024-04-24) for statistical analysis. OpenAI's ChatGPT was used for coding assistance.

## RESULTS

### Study Description

Data were collected from 225 adults with PaHIV aged 18–40 years. An overview of participant characteristics at the time of evaluation is presented in Table 1. Most study participants identified as Black (86%) and female (55%). The median age was 27 (IQR 23, 30) years. One hundred eighty-four (83%) were virally suppressed, with an HIV viral load <50 copies/mL. Thirty-nine (18%) were viremic, with a median viral load of 470 (IQR 121, 4090) copies/mL at the time of data capture. The median absolute CD4+ T cell count was 634 (IQR 438, 815) cells/μL, and the median duration on ART was 19 (IQR 13, 22) years. At the time of assessment, 219 (97%) of participants received regimens containing NRTIs (Table 1). The most common ART regimen included an integrase inhibitor (INSTI), and a total of 47 (21%) individuals were currently receiving a boosted PI regimen.

**Table 1.** Characteristics of the Study Population

Characteristics	All (n = 225)	WHO Hypertensive (n = 21)	AHA Hypertensive (n = 48)
Age, y	27 (23–30)	30 (27–33)	28.5 (26–31)
Gender			
Female	123 (55)	11 (52)	26 (54)
Male	101 (45)	10 (48)	22 (46)
Unspecified	1 (0.4)	…	…
Black ethnicity	189 (86)	16 (80)	41 (87)
Blood pressure investigations			
ECG	3 (1)	3 (14)	3 (6)
Echocardiography	5 (2)	5 (24)	5 (10)
Albumin:creatinine ratio	9 (4)	7 (33)	8 (17)
Antihypertensive medications	9 (4)	9 (43)	9 (19)
BMI kg/m^2^	25.5 (22.5–30.3)	28 (24–33)	27 (23–33)
BMI ≥ 25 kg/m^2^	121 (55)	13 (62)	31 (65)
Smoker	52 (24)	7 (33)	16 (33)
Absolute CD4 T + cells, cells/mm^3^	634 (438–815)	692 (362–862)	632 (380–787)
Nadir CD4 T + cells, cells/mm^3^	280 (65–459)	210 (45–340)	158 (29–320)
Current HIV viral load			
<50 copies/mL	184 (83)	18 (86)	41 (85)
50–400 copies/mL	19 (9)	1 (5)	2 (4)
>400 copies per/mL	20 (9)	2 (10)	5 (10)
Years on ART	19 (13–22)	20 (15–24)	21.5 (17–25)
Current ART			
NRTI	219 (97)	21 (100)	48 (100)
NNRTI	24 (11)	1 (5)	2 (4)
Abacavir	49 (22)	3 (14)	11 (23)
TAF	139 (62)	12 (57)	28 (58)
PI	47 (21)	4 (19)	9 (19)
INSTI	164 (73)	18 (86)	39 (81)
Previous ART			
Abacavir	50 (22)	8 (38)	17 (35)
TAF	7 (3)	2 (10)	3 (6)
PI	71 (32)	11 (52)	25 (52)
INSTI	21 (9)	2 (10)	6 (13)
Lipids			
Total cholesterol, mmol/L	4.30 (3.80–4.90)	4.30 (3.98–5.00)	4.30 (3.85–5.00)
Triglycerides, mmol/L	0.80 (0.60–1.28)	0.91 (0.62–1.63)	0.83 (0.60–1.40)
HDL-C, mmol/L	1.31 (1.10–1.51)	1.22 (0.99–1.56)	1.28 (1.0–1.54)
LDL-C, mmol/L	2.60 (2.08–3.00)	2.70 (2.17–3.25)	2.70 (2.00–3.28)
Non-HDL, mmol/L	3.00 (2.50–3.50)	3.10 (2.55–3.83)	3.10 (2.50–3.85)
HbA1C, mmol/mol	35 (31–37)	35 (32–37)	35 (32–37)
Metabolic syndrome	6 (3)	4 (20)	6 (13)

Data are median (IQR) or n (%). Ethnicity data were unavailable for four participants. Metabolic syndrome is defined as triglycerides ≥ 1.7 mmol/L, HDL levels <1.04 mmol/L (men) and <1.29 mmol/L (women), and BP ≥130/85 mmHg.

Abbreviations: AHA, American Heart Association; ART, antiretroviral therapy; BMI, body mass index; ECG, electrocardiogram; HbA1C, glycated hemoglobin; HDL, high-density lipoprotein; INSTI, integrase inhibitor; LDL, low-density lipoprotein; NNRTI, non-nucleoside reverse transcriptase inhibitor; NRTI, nucleoside reverse transcriptase inhibitor; PI, protease inhibitor; TAF, tenofovir alafenamide; WHO, World Health Organization.

### Prevalence of WHO-defined Hypertension and Associated Factors

Twenty-one (9%) of the participants met the criteria for WHO-defined hypertension (Table 1). Of those, 62% had a BMI ≥25, and 20% met the criteria for metabolic syndrome. Nine (43%) individuals were receiving antihypertensive medication. Participants with WHO-defined hypertension were significantly older than those without hypertension (*P* = .01) (Table 2). Of the 21 individuals with hypertension, 11 (52%) had previously received a PI-containing ART regimen, which was a significantly higher percentage compared to those who were not hypertensive (*P* = .03). There were also significantly more individuals with past AIDS-defining illnesses in the hypertensive group compared with the non-hypertensive group (*P* = .01).

**Table 2.** Predictors of Hypertension

	WHO			AHA		
Characteristics	Hypertensive (n = 21)	Non-hypertensive (n = 204)	*P* Value	Hypertensive (n = 48)	Non-hypertensive (n = 177)	*P* Value
Age	30 (27–33)	27 (23–30)	.01	28.5 (26–31)	26 (22–30)	.01
BMI kg/m^2^	27.9 (23.6–32.9)	25.5 (22.3–29.9)	.13	26.9 (23.2–33.0)	25.3 (22.3–28.8)	.07
Smoker	7 (33)	45 (23)	.30	16 (33)	36 (21)	.07
Nadir CD4 T cells, cells/mm^3^	210 (45–340)	281 (66–476)	.20	158 (29–320)	290 (90–516)	.01
Y on ART	20 (15–24)	19 (13–22)	.20	22 (17–25)	18 (13–22)	<.01
Previous ART						
Abacavir	8 (38)	42 (21)	.09	17 (35)	33 (19)	.01
PI	11 (52)	60 (29)	.03	25 (52)	46 (26)	<.01
Past AIDS-defining illness	8 (38)	30 (15)	.01	14 (29)	24 (14)	.01
Metabolic syndrome	4 (20)	2 (1)	<.01	6 (13)	0 (0)	<.01

Data are median (IQR) or n (%). *P* values were calculated using the Wilcoxon rank sum test for continuous data, Pearson's Chi-squared test and Fisher's exact test for categorical data.

Abbreviations: AHA, American Heart Association; AIDS, acquired immunodeficiency syndrome; ART, antiretroviral therapy; BMI, body mass index; PI, protease inhibitors; WHO, World Health Organisation.

### Prevalence of AHA-defined Hypertension and Associated Factors

According to AHA guidelines, 48 (21%) individuals had hypertension (Table 1). About 65% were overweight, with a BMI ≥25, and 13% met the criteria for metabolic syndrome. Nine (19%) individuals were receiving antihypertensive medication. Participants with AHA-defined hypertension were significantly older than those without hypertension (*P* = .01) (Table 2). Nadir CD4+ T cell count, years on ART, and past AIDS-defining illnesses were all significantly different when comparing the hypertensive group to the non-hypertensive group (*P* = .01, *P* < .01, and *P* = .01, respectively). In the hypertensive group, 25 (52%) individuals had previously received a PI-containing ART regimen, compared twitho 46 (26%) individuals in the non-hypertensive group (*P* < .01). In addition, prior exposure to abacavir was significantly more prevalent in the AHA-defined hypertensive group relative to normotensive participants (*P* = .01).

### Prevalence of Metabolic Syndrome and Association With Hypertension

Among the study cohort, 6 (3%) met the criteria for metabolic syndrome (Table 1). The prevalence of metabolic syndrome was significantly higher among participants with hypertension, regardless of the diagnostic definition used (Table 2). Specifically, 4 participants with hypertension as defined by WHO met criteria for metabolic syndrome, compared to 2 in the normotensive group. Similarly, among those classified as hypertensive using AHA criteria, 6 individuals had metabolic syndrome, also significantly more than in the non-hypertensive group.

### Prevalence of PDAY Risk Scores and Associated Factors

**Figure 1.**
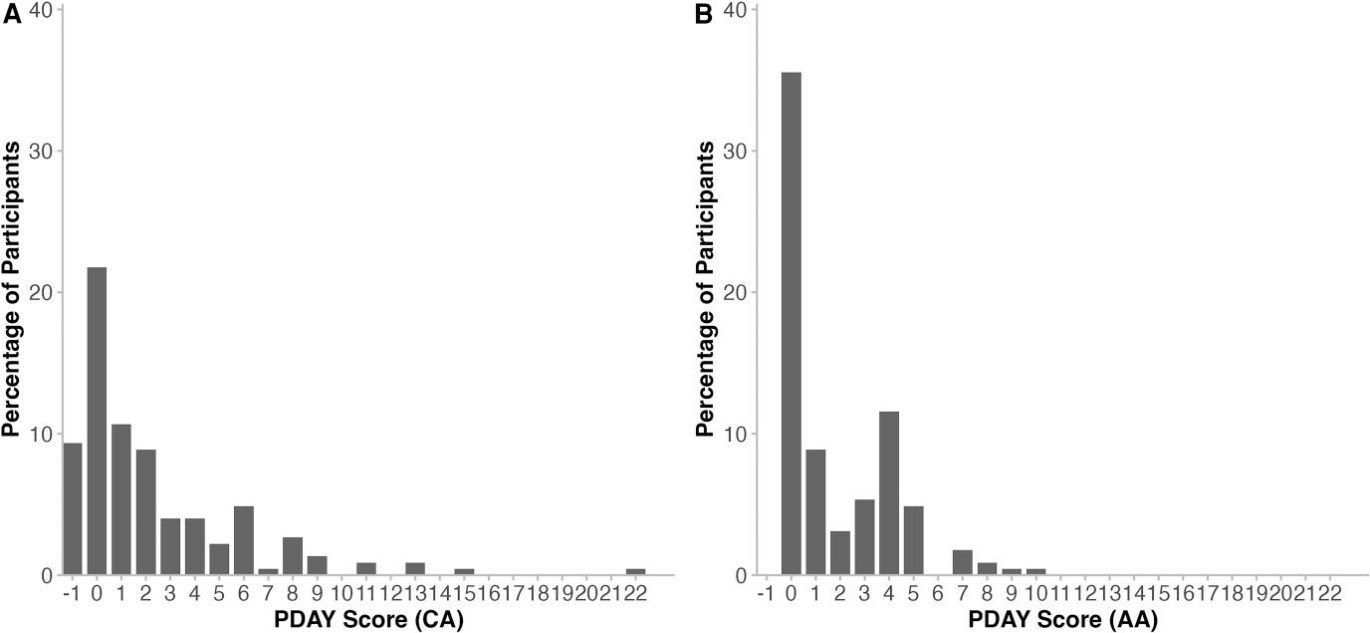
Distribution of pathobiological determinants of atherosclerosis in youth (PDAY) risk scores. *A*, Distribution of coronary arteries (CA) PDAY risk scores. *B*, Distribution of abdominal aorta (AA) PDAY risk scores.

The distribution of CA and AA PDAY scores is presented in Figure 1 and Table 3. Of the 225 individuals in the study, 162 (72%) had complete data to calculate CA and AA PDAY risk scores (Table 4). For CA, 92 (57%) had scores ≥1. For AA, 82 (51%) had scores ≥1. In univariable analyses, potential predictors of a high CA PDAY risk score (≥1) at a significance level of *P* < .10 included male sex, current HIV viral load, current CDC category, current use of a boosted PI, ever use of a boosted PI, and current lamivudine use. For AA PDAY risk scores, potential predictors were male sex, current HIV viral load, CDC category, and ever use of a boosted PI. Due to a moderate correlation between current use of boosted PI and ever use of PI, only ever use of boosted PI, which had the highest c-statistic, was retained in multivariable modeling. In the multivariable logistic regression model for CA, male sex, CDC category C and ever use of boosted PI remained significant at a significance level of *P* ≤ .05 (Table 5). For AA, current HIV viral load >5000 copies/mL and CDC category C were significantly associated with a PDAY score ≥1.

**Table 3.** Distribution of Modifiable Atherosclerotic Risk Factors Included in the Pathobiological Determinants of Atherosclerosis in Youth (PDAY) Scoring System Among the 225 Adults With Perinatally Acquired HIV in the Study Population at Their Most Recent Visit

Risk Factor	Coronary Arteries Points	Abdominal Aorta Points	n	(%)
Modifiable risk factors				
Non-HDL cholesterol, mg/dL				
<130	0	0	147	(70)
130–159	2	1	42	(20)
160–189	4	2	13	(6)
190–219	6	3	7	(3)
≥220	8	4	2	(1)
HDL cholesterol, mg/dL				
<40	1	0	32	(15)
40–59	0	0	129	(62)
≥60	−1	0	48	(23)
Smoking				
Nonsmoker	0	0	168	(76)
Smoker	1	4	52	(24)
Blood pressure				
Normotensive	0	0	204	(91)
Hypertensive	4	3	21	(9)
Obesity (BMI), kg/m^2^				
Male				
≤30	0	0	80	(36)
>30	6	0	19	(8)
Female				
≤30	0	0	82	(37)
>30	0	0	39	(17)
Glycemia				
Not hyperglycemic	0	0	174	(99)
Hyperglycemic	5	3	1	(1)

Abbreviations: BMI, body mass index; HDL, high-density lipoproteins; PDAY, pathobiological determinants of atherosclerosis in youth.

**Table 4.** Univariable Predictors of Pathobiological Determinants of Atherosclerosis in Youth (PDAY) Risk Scores Among the 162 Adults With Perinatally Acquired HIV in the Study Population at Their Most Recent Visit

	Coronary Arteries PDAY Score				Abdominal Aorta PDAY Score			
Characteristic	≤ 0 (n = 70)	≥ 1 (n = 92)	*P* value	C-statistic	≤ 0 (n = 80)	≥ 1 (n = 82)	*P* value	C-statistic
Age, y, n (%)	…	…	0.11	…	…	…	0.20	…
18–19	4 (6)	4 (4)	…	…	5 (6)	3 (4)	…	…
20–24	30 (43)	29 (32)	…	…	34 (43)	25 (30)	…	…
25–29	24 (34)	28 (30)	…	…	25 (31)	27 (33)	…	…
≥30	12 (17)	31 (34)	…	…	16 (20)	27 (33)	…	…
Female, n (%)	49 (70)	43 (47)	<0.01	0.62	51 (64)	41 (50)	0.08	0.57
Current HIV viral load, copies/mL, n (%)	…	…	0.05	0.56	…	…	0.01	0.57
≤400	67 (97)	77 (85)	…	…	77 (97)	67 (83)	…	…
401–5000	1 (1)	7 (8)	…	…	1 (1)	7 (9)	…	…
>5000	1 (1)	7 (8)			1 (1)	7 (9)		
Missing	1 (1)	1 (1)			1 (1)	1 (1)		
Current CDC category, n (%)			.02	0.62			.03	0.62
A	29 (47)	23 (29)			32 (45)	20 (28)		…
B	13 (21)	13 (16)			15 (21)	11 (15)		…
C	20 (32)	44 (55)			24 (34)	40 (56)		…
Missing	8 (11)	12 (13)			9 (11)	11 (13)		…
ART								
Current use, n (%)	69 (99)	92 (100)	.4	…	79 (99)	82 (100)	.50	…
Boosted protease inhibitor								
Current use, n (%)	9 (13)	22 (24)	.08	0.56	12 (15)	19 (23)	.20	…
Ever use, n (%)	24 (34)	59 (64)	<.01	0.65	33 (41)	50 (61)	.01	0.60
Cumulative duration of use, years, median (q1, q3)	6.6 (3.5, 10.7)	6.5 (3.3, 9.0)	.70	…	6.5 (3.2, 10.5)	6.6 (3.7, 9.2)	>.90	…
Ritonavir	…	…		…	…	…		…
Current use, n (%)	2 (3)	7 (8)	.30	…	5 (6)	4 (5)	.70	…
Abacavir	…	…		…	…	…		…
Current use, n (%)	18 (26)	18 (20)	.40	…	19 (24)	17 (21)	.60	…
Lamivudine								
Current use, n (%)	24 (34)	19 (21)	.05	0.57	25 (31)	18 (22)	.20	…
Tenofovir disoproxil fumarate								
Ever use, n (%)	6 (9)	11 (12)	.50		8 (10)	9 (11)	.80	

Data are median (IQR) or n (%). *P* values were calculated using the Wilcoxon rank sum test for continuous data, Pearson's Chi-squared test and Fisher's exact test for categorical data.

Abbreviations: A, not/mildly symptomatic; ART, antiretroviral therapy; B, moderately symptomatic; BMI, body mass index; C, severely symptomatic (AIDS definition); CDC, Centers for Disease Control and Prevention; HDL, high-density lipoproteins; PDAY, pathobiological determinants of atherosclerosis in youth.

**Table 5.** Predictors of Coronary Arteries and Abdominal Aorta Pathobiological Determinants of Atherosclerosis in Youth (PDAY) Risk Scores ≥1 Versus ≤0 Among the 162 Adults With Perinatally Acquired HIV in the Study Population at Their Most Recent Visit

	Coronary Arteries		Abdominal Aorta	
Characteristics	Multivariable Odds Ratio (95% CI)	*P*	Multivariable Odds Ratio (95% CI)	*P*
Sex	…	.02	…	.12
Male	2.46 (1.17, 5.32)		1.77 (.86, 3.66)	
Female	Reference		Reference	
Current HIV viral load, copies/mL	…	.13	…	.03
≤400	Reference		Reference	
401–5000	2.98 (.35, 67.40)		4.75 (.64, 99.0)	
>5000	6.94 (.88, 150.00)		8.85 (1.29, 180.00)	
Current CDC category	…	.02	…	.03
A	Reference		Reference	
B	1.45 (.52, 4.09)		1.44 (.53, 3.95)	
C	3.11 (1.37, 7.36)		2.87 (1.30, 6.55)	
Ever boosted protease inhibitor use	…	.02	…	.16
Yes	2.48 (1.14, 5.49)		1.66 (.82, 3.42)	
No	Reference		Reference	
Current lamivudine use	…	.49	…	
Yes	0.74 (.318, 1.72)	…		
No	Reference		…	

Current is defined as the most recent measurement.

Abbreviations: A, not/mildly symptomatic; B, moderately symptomatic; C, severely symptomatic (AIDS definition); CDC, Centers for Disease Control and Prevention.

## DISCUSSION

In this review of 225 adults with lifelong HIV (median age 27 years), the prevalence of WHO- and AHA-defined hypertension was broadly similar to that reported in age-matched general population studies from the UK and US, respectively. Importantly, over half of participants had elevated PDAY scores, indicating early subclinical atherosclerotic risk despite their young age and good virological control. These findings suggest that traditional CVD risk scores may underestimate risk in this population and that PDAY may provide a more relevant measure of early cardiovascular risk in adults with PaHIV. In addition, whilst most national guidelines recommend commencing statin therapy for all people living with HIV over the age of 40, the increased PDAY scores in this group of younger adults with PaHIV suggest this recommendation may need to be extended to younger people in this setting.

Several previous studies have reported an increased risk of CVD and its risk factors among people with HIV compared to the age-matched general population [21–23]. Zoest et al. [24] found a higher prevalence of hypertension among people with HIV, when compared to those without HIV. The median age was 52.9 years for those with HIV and 52.2 years for those without HIV. After adjusting for age, sex, ethnicity, family history of hypertension, smoking, alcohol use, physical activity, and BMI, the association remained significant. Our findings did not corroborate this effect, likely due to the younger age of our cohort and shorter cumulative exposure compared with the older population in Zoest et al.

Age is a well-recognized independent risk factor for the development of CVD [25, 26]. Hypertension, a primary risk factor for CVD, becomes more prevalent with age due to mechanisms like oxidative stress, inflammation, and endothelial dysfunction [27, 28]. This aligns with our findings; individuals in the hypertensive group were significantly older, had lived longer with HIV, with a greater exposure to ART, and higher rates of prior AIDS-defining illnesses. This likely reflects a cohort effect, as older participants born in the pre-ART era, initiated ART later in life and may have experienced prolonged periods of immunosuppression. As younger individuals age on more effective ART regimens, such as INSTIs, their long-term cardiovascular risk profile may differ. This may also explain why metabolic syndrome was rare in our cohort despite more than half having elevated PDAY scores: metabolic syndrome requires multiple concurrent abnormalities and is uncommon at this age, whereas PDAY captures early subclinical risk. Missing data on glucose and waist circumference may also have underestimated metabolic syndrome prevalence.

PIs have been linked to insulin resistance and dyslipidemia, risk factors for hypertension and hypercholesterolemia [29]. A study by Byonanebye et al. [30] examined the association between hypertension and various ART regimens. The analysis included 4606 individuals from RESPOND, a consortium of 17 observational cohorts tracking 32 085 people with HIV. Both INSTI-based and PI-based ART regimens were associated with a higher risk of hypertension compared to NNRTIs. Crane et al. [31] identified an association between hypertension and INSTI-based regimens, but this was not significant after adjusting for BMI. Unlike Byonanebye et al. and Crane et al., we did not observe significant associations with INSTI exposure, though prior PI use was more common among hypertensive participants.

Among individuals with AHA-defined hypertension, nadir CD4+ T cell counts were significantly lower than those in the non-hypertensive group, and years on ART were significantly longer. Manner et al. reported similar findings, with a nadir CD4+ T cell count <50 cells/µL and ART duration as significant predictors of hypertension [32]. In our study, past AIDS-defining illnesses were also significantly more prevalent in both the WHO- and AHA-defined hypertensive groups, likely reflecting lower nadir CD4+ counts. This potentially reflects a broader pattern, where older individuals experienced a different virological and treatment landscape, having initiated ART later, with lower nadir CD4+ T cell counts and a higher incidence of opportunistic infections. However, it could also reflect survival bias, where individuals with slower HIV progression survive long enough to develop hypertension, despite having had prior AIDS-defining conditions. In contrast, younger individuals typically started ART earlier, often at diagnosis, with better immune preservation. These differences may contribute to cardiovascular risk beyond age alone. It is also worth noting that a large proportion of our cohort is of Black ethnicity, a group known to have higher hypertension and CVD risk than the general UK and US populations [33, 34]. Current algorithms may not fully capture these elevated baseline risks, potentially underestimating cardiovascular risk.

Elevated PDAY risk scores were observed in 57% of individuals for CA and 51% for AA, indicating a substantial burden of subclinical atherosclerotic risk. These proportions exceeded those reported by Patel et al. [11] in a younger PaHIV cohort, likely reflecting age-related atherosclerosis progression, HIV-associated inflammation, or cumulative ART exposure. In multivariable analysis, male sex, CDC category C, and ever use of boosted PIs were significantly associated with elevated CA scores, while current HIV viral load >5000 copies/mL and CDC category C were associated with elevated AA scores. Patel et al. also identified immunosuppression and PI exposure as predictors, consistent with our findings. The overlap in predictors underscores their relevance to early cardiovascular risk in people with HIV, while differences in age and treatment history may explain the higher prevalence observed in our study. These findings support early cardiovascular risk assessment and ongoing monitoring, particularly among adults with advanced HIV disease or prolonged exposure to older regimens. The recent REPRIEVE trial demonstrated that statins significantly reduced major cardiovascular events in people with HIV over 40, even in those with low to moderate cardiovascular risk [35]. However, individuals with PaHIV were omitted, and most of our cohorts have not yet reached statin eligibility age. This raises important questions on how best to assess and manage early cardiovascular risk in younger people with HIV, particularly those with PaHIV, who may already carry substantial long-term risk.

Emerging evidence also supports the use of non-traditional assessment modalities, such as coronary artery stiffness imaging, for early detection of cardiovascular risk in people with HIV, particularly when conventional scores may underestimate risk [36]. In parallel, growing insights into the underlying mechanisms, including immune activation, endothelial dysfunction, and inflammation, further underscore the complex interplay between HIV and CVD [22, 37].

There are several limitations to our study. Firstly, the cross-sectional design allows assessment of associations but precludes causal inference between HIV, ART exposure, and cardiovascular risk. Longitudinal analysis was not possible due to the retrospective nature of the dataset, as clinical records did not provide standardized time points across participants. Prospective longitudinal studies are needed to understand long-term cardiovascular outcomes in individuals with PaHIV. Secondly, reliance on retrospective data may affect accuracy and completeness, potentially leading to misclassification or underreporting of hypertension and key variables. Missing or incomplete records could also introduce bias. While inclusion of PDAY scores strengthens the study by providing validated estimates of subclinical atherosclerotic burden, these were available only for a subset of participants, limiting statistical power and generalizability. Baseline characteristics were broadly similar between those included and excluded from PDAY analysis, though excluded individuals tended to be older and less likely to have BMI >30, suggesting that the included group is reasonably representative, although selection bias cannot be ruled out (Supplementary Table 1). For some individuals, complete data on exposure to certain older antiretrovirals, including stavudine (d4T) and didanosine (ddI), were unavailable, despite their known metabolic risks. Smoking status was recorded as a binary variable, limiting the cumulative exposure assessment. The small size of the subgroups with metabolic syndrome limits the robustness of these estimates. Lastly, lifestyle-related factors such as physical activity, alcohol use, diet, and family history of hypertension were not captured, which limits interpretation of associations. In the UK, young people with PaHIV usually transition to adult HIV care between 16 and 18 years through structured pathways and joint pediatric–adult clinics. Despite this support, transition remains a vulnerable period with risks of adherence difficulties, psychosocial stress, and disengagement, which are important when interpreting outcomes in our adult cohort. The generalizability of our findings is limited by the demographic and healthcare context of this cohort, which was predominantly Black, relatively young, and managed in a UK NHS setting with universal ART access. Results may therefore not extend to older PaHIV populations or those in resource-limited settings, where most adults with PaHIV are younger. Our cohort also includes some of the earliest survivors, who were exposed to periods of viremia in childhood before pediatric formulations and wider availability of ART.

In conclusion, adults with PaHIV in our cohort exhibited a substantial burden of subclinical cardiovascular risk, with over half showing elevated PDAY scores and a notable minority meeting criteria for hypertension or metabolic syndrome. These results highlight the importance of early cardiovascular monitoring and tailored preventative strategies for this population, particularly as they age on lifelong ART and the potential to offer statin therapy to reduce the CVD risk at a younger age.

## Supplementary Material

ofaf629_Supplementary_Data

## Supplementary Data

Supplementary materials are available at *Open Forum Infectious Diseases* online. Consisting of data provided by the authors to benefit the reader, the posted materials are not copyedited and are the sole responsibility of the authors, so questions or comments should be addressed to the corresponding author.
