# Supplementary material for: Evaluation of Cardiovascular Risk Factors Among Adults With Perinatally Acquired HIV
Source: Open Forum Infect Dis. 2025 Nov 6;12(11):ofaf629. doi: 10.1093/ofid/ofaf629 (PMC12596388; doi:10.1093/ofid/ofaf629)
Supplement: ofaf629_Supplementary_Data [file ofaf629_supplementary_data.docx]

**Supplementary Table 1: Characteristics of participants included and excluded from PDAY score analysis**

Data are n (%). BMI, Body Mass Index.

*Data were only available for 56 individuals
